# Activity-Dependent Plasticity of Astroglial Potassium and Glutamate Clearance

**DOI:** 10.1155/2015/109106

**Published:** 2015-08-04

**Authors:** Giselle Cheung, Jérémie Sibille, Jonathan Zapata, Nathalie Rouach

**Affiliations:** ^1^Neuroglial Interactions in Cerebral Physiopathology, Center for Interdisciplinary Research in Biology, Collège de France, CNRS UMR 7241, INSERM U1050, Labex Memolife, PSL Research University, 75005 Paris, France; ^2^Université Paris Diderot, Sorbonne Paris Cité, 75013 Paris, France

## Abstract

Recent evidence has shown that astrocytes play essential roles in synaptic transmission and plasticity. Nevertheless, how neuronal activity alters astroglial functional properties and whether such properties also display specific forms of plasticity still remain elusive. Here, we review research findings supporting this aspect of astrocytes, focusing on their roles in the clearance of extracellular potassium and glutamate, two neuroactive substances promptly released during excitatory synaptic transmission. Their subsequent removal, which is primarily carried out by glial potassium channels and glutamate transporters, is essential for proper functioning of the brain. Similar to neurons, different forms of short- and long-term plasticity in astroglial uptake have been reported. In addition, we also present novel findings showing robust potentiation of astrocytic inward currents in response to repetitive stimulations at mild frequencies, as low as 0.75 Hz, in acute hippocampal slices. Interestingly, neurotransmission was hardly affected at this frequency range, suggesting that astrocytes may be more sensitive to low frequency stimulation and may exhibit stronger plasticity than neurons to prevent hyperexcitability. Taken together, these important findings strongly indicate that astrocytes display both short- and long-term plasticity in their clearance of excess neuroactive substances from the extracellular space, thereby regulating neuronal activity and brain homeostasis.

## 1. Introduction

Astrocytes, the most abundant cell type of the brain, have been considered as the active players in the tripartite synapse with neurons. A wide variety of physiological functions of astrocytes have been identified ranging from structural and metabolic support to the modulation of synaptic transmission and information processing. They have also been found to play major roles in both the progression and repair of CNS pathologies like inflammation, epilepsy, ischemia, neurodegenerative diseases, and neurodevelopmental disorders [1–3]. Recently, it has been established that astrocytes actively influence neuronal plasticity and memory formation [4–9]. However, the reciprocal phenomenon involving astroglial plasticity in response to changes in neuronal activity has been less well explored and understood. Indeed, it has been demonstrated that astrocytes display both short- and long-term plasticity similar to neurons [10–22]. This review focuses on the ability of astrocytes to regulate extracellular levels of signalling molecules, particularly potassium (K^+^) and glutamate, in a neuronal activity-dependent manner. The expression, properties, and regulations of the important cellular components will first be introduced. Then, we will present and discuss important research findings demonstrating plastic modulations of these astroglial properties in response to changes in physiological neuronal activities.

## 2. Molecular Machinery for Astroglial Potassium and Glutamate Clearance

### 2.1. Extracellular Potassium and Glutamate Levels

K^+^ and glutamate are essential and abundant neuroactive molecules participating in excitatory synaptic transmission of the brain. In particular, ~80% of neuronal K^+^ release originates from postsynaptic elements [23]. It has been reported that just a single action potential can elevate the resting level of extracellular K^+^ from 3 to 4 mM [24]. This increase can also be stronger under conditions in which the extracellular space volume is decreased. During hyperactivity, this level can go up to 10 to 12 mM [25]. Pathological situations can even transiently elevate extracellular K^+^ level to >30 mM [26]. The excitatory neurotransmitter glutamate, on the other hand, is released via synaptic vesicles during excitatory synaptic activity and is not degraded extracellularly. Its removal highly depends on diffusion and transporter systems. While intracellular concentration of glutamate is about 10 mM [27], extracellular glutamate level varies considerably from one compartment to the other. In fact, a study revealed that each glutamatergic synaptic vesicle contains around 4,000 molecules of glutamate [28]. Thus, it is not surprising that glutamate concentration in the synaptic cleft can increase transiently from <20 nM to 1 mM following action potentials [29]. On the contrary, extrasynaptic glutamate level has been reported to vary over a large range from 0.02 to 30 *μ*M [30, 31]. Although this variability is partly due to limitations in assessment techniques used, it can also be explained by different glutamate clearance rates and mechanisms between perisynaptic and nonsynaptic compartments [32]. Given that a significant accumulation of both K^+^ and glutamate occurs during physiological neuronal transmission, it is crucial that their levels be tightly regulated to prevent hyperexcitability and excitotoxicity. Indeed, this important task is carried out predominantly and efficiently by neighboring astrocytes in the CNS [26, 27].

### 2.2. Potassium Homeostasis

K^+^ clearance by astrocytes is conducted either by net K^+^ uptake or by K^+^ spatial buffering [26, 33]. The first route largely involves the activity of cotransporters (Na^+^-K^+^-ATPase or Na^+^-K^+^-2Cl^−^), as well as K^+^ and Cl^−^ channels. As a result, K^+^ ions transiently accumulate inside astrocytes and are released once extracellular K^+^ level drops [26]. On the contrary, K^+^ spatial buffering allows uptake and redistribution of K^+^ from areas of high to low extracellular K^+^ concentrations, in which no significant net intracellular accumulation occurs. This later mechanism has been found to be more efficient than diffusion alone [34]. In fact, it has been proposed that a single elongated astrocyte can effectively redistribute K^+^ from one end to the other [35]. In Müller cells of the retina, K^+^ siphoning, a specialized form of K^+^ spatial buffering in which glial K^+^ channel distribution directs K^+^ clearance into the vitreous humor, was extensively studied and found to involve inward rectifying K^+^ (Kir) channels [33]. In general, seven major subfamilies of Kir channels have been identified (Kir1 to Kir7), each consisting of two transmembrane domains, an inner pore, and intracellular N- and C- termini [36]. They form tetrameric homomers and heteromers with different rectifying properties and conductances [37]. Their inward rectifying properties allow preferential passage of K^+^ ions into the cells. These channels are perfect candidates for regulation of K^+^ homeostasis as they have high open probability at glial resting membrane potentials and their conductance is variable relative to extracellular K^+^ concentrations [38]. Among the subfamilies, Kir2, Kir4, and Kir5 are most commonly studied in glial cells of the brain. In particular, Kir4.1 is highly abundant in astrocytes of different brain regions [39] and is enriched in astrocytic processes surrounding synapses and blood vessels [40]. In Müller cells, it was proposed that the strongly rectifying Kir2.1 subtype is uniformally expressed at plasma membranes, enabling K^+^ influx from the synaptic plexiform layers, while the weakly rectifying Kir4.1 subtype is responsible for the exit of K^+^ at the endfeet and lateral processes [41–43]. This specific compartmentalization strongly reflects regional specific functions and regulations of Kir channels.

### 2.3. Glutamate Clearance

As glutamate is not degraded upon release, cellular mechanisms must exist to quickly remove excess glutamate in order to avoid receptor desensitization and excitotoxicity. It was shown in the neonatal hippocampus that glutamate clearance depends primarily on diffusion, whereas glial transporter uptake dominates in juvenile mice [44]. This coincides with periods of synaptic maturation at which point extracellular volume significantly decreases rendering diffusion less effective. Indeed, the glutamate transporter systems have been identified to be an efficient way of removing extracellular glutamate.

The expression of excitatory amino-acid transporters (EAATs) has been extensively studied revealing that EAAT1/GLAST and EAAT2/GLT-1 are found mostly in glia while EAAT3/EAAC1, EAAT4, and EAAT5 are expressed in neurons [45]. EAATs can alternate between two conformations to transport glutamate inside the cells against its concentration gradients. This requires efficient cotransport of glutamate and ions (three Na^+^ and one H^+^) from the outside inwards and the counter-transport of one K^+^ ion from the inside to revert back to the outward conformation [46]. Crystal structures revealed that glutamate transporters are homomers containing three subunits [47, 48]. Different subtypes also display varying affinities for glutamate [45]. GLT-1 and GLAST are expressed early in development despite being at low levels [49]. This increases dramatically in the most active period of synaptogenesis (during the third and fourth postnatal weeks) and throughout adult life [50]. In the adult hippocampus, astroglial GLT-1 and GLAST are expressed at high amount and density (0.7 to 1.3 mM and up to 8,500 molecules per *μ*m^2^) [51].

Intriguingly, like Kir channels, it was determined that the concentrations of GLT-1 and GLAST on astroglial membrane vary considerably depending on neighboring structures. For example, membranes facing nerve terminals, axons, and dendritic spines have higher densities than those facing other astrocytes, cell bodies, or capillaries [52]. To study these transporters, robust glutamate transporter currents have been successfully measured and characterized using electrophysiological techniques in hippocampal CA1 astrocytes [53, 54]. Furthermore, GLT-1 and GLAST are believed to account for most of the glutamate uptake conducted by all EAATs subtypes in mammalian CNS, further suggesting the importance of glial glutamate removal [27]. In terms of regional specific expression, GLT-1 is mostly expressed in the forebrain and GLAST in the cerebellum, inner ear, circumventricular organs, and the retina [27]. Consequently, mice lacking GLT-1 show lethal spontaneous seizures and are more susceptible to acute forebrain injury [55]. On the other hand, those lacking GLAST have increased susceptibility to injury to the cerebellum and experience impaired motor coordination [56].

### 2.4. Gap-Junctional Mediated Potassium and Glutamate Clearance by Astrocytes

Apart from the expression of K^+^ channels and glutamate transporters, networks of astrocytes are also known to siphon extracellular molecules from areas of high to low concentrations via gap-junction channels [9]. Typically, astrocytes express two connexin (Cx) subtypes, Cx43 and Cx30. Cx43 is expressed early, while Cx30 later in development [57]. Gap junction channels are formed across the membranes of two cells by two opposing hemichannels each composed of six Cx subunits [58]. They form channels allowing intracellular transfer of low molecular-weight molecules (<1.5 kDa). Thus, molecules like K^+^ and glutamate can be efficiently passed from cell to cell greatly facilitating extracellular clearance. Gap-junctional coupling among astrocytes plays significant roles in both net K^+^ uptake [59] and K^+^ spatial buffering [60, 61], as well as glutamate clearance [62, 63]. This mechanism offers an additional pathway for excess K^+^ ions and glutamate to be efficiently redistributed within networks of astrocytes. In various pathologies; however, the propagation of neurotoxic substances away from the site of injury has been thought to be both protective and destructive [2, 64]. Interestingly, when astrocytic gap-junctional coupling was reduced by blocking Cx43, a decrease in GLT-1 expression was also observed, suggesting possible regulatory roles of gap-junctional coupling in glial transporter activities [65].

## 3. Functions of Astroglial Potassium and Glutamate Clearance and Their Regulations

Like many other ion channels, Kir channels in the CNS have been linked to many processes of the brain like ontogenesis, regulation of hormone secretion, neurotransmitter release, control of seizure activity, and regulation of cerebral blood flow [66]. The properties regulating astroglial K^+^ clearance like channel activities and conductances are modulated by many factors. This includes changes in extracellular volume, pH, intracellular calcium (Ca^2+^) and Cl^−^ concentrations, and neurotransmitter release [26]. In addition, the opening of Kir channels is also sensitive to various types of extracellular and intracellular blockade. For examples, they are blocked by intracellular magnesium and polyamines as well as extracellular barium (Ba^2+^) ions in a voltage-dependent manner [67]. They are also blocked dose-dependently by cesium and tetraethylammonium [68]. Other studies have shown that they can also be activated by intracellular G-protein subunits [69] or adenosine triphosphate [70]. Interestingly, the water channel aquaporin 4 has been found to be strongly colocalized with Kir4.1 channels in Müller cells, possibly to prevent osmotic imbalance [71]. They are also coregulated by the same dystrophin-associated protein complexes intracellularly for subcellular distribution and clustering [72]. Astroglial conditional knockout lacking Kir4.1 induced in hippocampal astrocytes a severe depolarization [73] and in neurons enhanced responses to prolonged repetitive and to tetanic stimulations of Schaffer collaterals (SCs) [21]. These mice were also found to have severe ataxia and stress-induced seizures and even die prematurely [73].

The functional roles of glutamate transporters are diverse and vary among synapses. Although glutamate transporters are expressed in different cell types in the CNS, astrocytes are responsible for the majority of glutamate uptake and homeostasis [27]. Studies in the hippocampus have shown that while these transporters can affect the fast component of the synaptic glutamate transient via AMPA and NMDA receptors, they can also affect the slower components mediating spillover [45]. In addition, both neuronal and glial transporters have been found to limit the glutamate spillover, reinforcing synapse independence. Interestingly, synaptic plasticity could also be differentially influenced by the expression and activity of glial glutamate transporters. Indeed, it was demonstrated that long-term potentiation (LTP), but not long-term depression (LTD), was impaired in the hippocampal CA1 region of GLT-1 mutants [74]. In the amygdala, inhibition of glutamate transporters resulted in a loss of input specificity of LTP important for fear conditioning [75]. Behavioral defects like anhedonia and impaired spatial memory have also been observed after blocking astroglial GLT-1 activities [76]. Using GLT-1 inducible knockout mice, a recent study has demonstrated that the resulting increase in synaptic excitability was responsible for pathological repetitive behaviours [77].

There has been* in vitro* evidence indicating that coculturing astrocytes with neurons increases the expression of GLT-1 and GLAST [78, 79]. In addition, several posttranslational regulations of glutamate transporters have also been demonstrated. Phosphorylation by protein kinase C (PKC) has been found to modulate the transport activity of GLAST [80], as well as cell-surface expression of GLT-1 [81, 82]. It was also observed that sumoylated GLT-1 is translocated to intracellular compartments [83]. Furthermore, glutamate transporters possess an SH-based redox regulatory mechanism [84] and may be regulated by serum- and glucocorticoid-inducible kinases [85]. Arachidonic acid has also been shown to inhibit GLT-1 activity [86]. In addition, the cell-surface expression of GLAST in cultured astrocytes is rapidly upregulated by glutamate binding, suggesting modulations in transporter trafficking [87]. Interestingly, it was proposed that neuronal activity could modulate the distribution of GLT-1 clusters in developing astrocytes [88]. Finally, processes like regulated glial endo-/exocytosis [89–91] and surface diffusion [92] could also significantly account for differential surface expression of glial glutamate transporters.

It is conceivable that expression levels, localization, and properties of glial channels and transporters largely determine the efficiency of K^+^ and glutamate clearance from extracellular space, thereby modulating synaptic plasticity. For this reason, relevant molecular components must be tightly regulated for optimal expression, distribution, and activities.

## 4. Electrophysiological Methods to Study K^+^ and Glutamate Transporter Currents in Astrocytes

**Figure 1:**
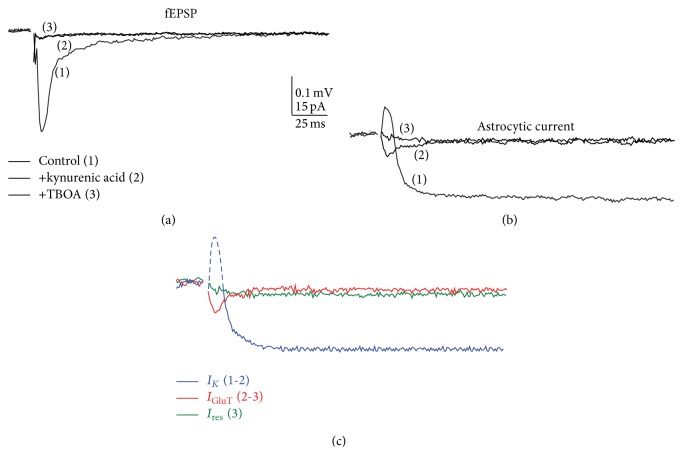
Pharmacological isolation of astroglial currents. Dual patch clamp recordings revealed synaptically evoked astroglial currents in hippocampal CA1 region. Sample traces are shown for simultaneously recorded (a) neuronal field excitatory postsynaptic potentials (fEPSPs) and (b) astroglial currents induced by a single stimulation of Schaffer collaterals. This was carried out under control conditions (100 *μ*M picrotoxin; 1), and subsequent addition of an ionotropic glutamate receptor antagonist (5 mM kynurenic acid; 2), and a glutamate transporter antagonist (200 *μ*M TBOA; 3). (c) Sample traces of pharmacologically isolated astroglial currents are shown. Astrocytic potassium current was isolated by subtracting the kynurenic acid-insensitive component from the total current (*I*_*K*_, 1-2, blue). Note that the initial fast outward current component (dash blue) reflects fEPSPs generated by adjacent pyramidal cells, while *I*_*K*_ corresponds to the slow inward current (solid blue). The kynurenic acid insensitive current (2) consists of a fast inward current and a slow residual component of smaller amplitude; the latter was isolated by subsequent application of TBOA (*I*_res_, 3, green). Subtraction of *I*_res_ from the response in the presence of kynurenic acid resulted in the glutamate transporter current component (*I*_GluT_, 2-3, red). Scale bars = 0.1 mV for fEPSPs, 15 pA for astroglial currents; 25 ms. This figure (a–c) is extracted from [21].

Over the years, dual electrophysiological recordings in acute hippocampal slices have allowed the identification and pharmacological dissection of synaptically-evoked astroglial currents while monitoring neuronal plasticity [4, 21, 93]. A complex astroglial biphasic response evoked by single stimulation of SCs (0.05 Hz) from* stratum radiatum* hippocampal astrocytes has been identified and it consisted of a fast outward current, reflecting the extracellular field potential generated by receptors on CA1 pyramidal neurons, followed by a slow inward current persisting for several seconds (Figure 1). This long-lasting K^+^ current mediated by Kir4.1 channels constitutes ~80% of the total astrocytic currents and can be isolated in the hippocampus by addition of kynurenic acid (Figure 1(c), *I*_*K*_), an ionotropic glutamate receptor antagonist, which has no direct effect on hippocampal passive astrocytes connected by gap junctions and lacking glutamate ionotropic receptors [21]. In addition, a transient glutamate transporter current (*I*_GluT_) can then be isolated by subsequent addition of glutamate transporter antagonists DL-threo-*β*-benzyloxyaspartic acid (TBOA) and dihydrokainate (DHK) [6, 21, 53, 93–96]. Finally, a slow residual current (*I*_res_) mediated by GABA transporters (GATs) and Kir4.1-independent K^+^ channels remains [21]. Interestingly, such astrocytic currents increased with neuronal input over a wide range of fiber volley amplitudes, suggesting that glial cells are reliable sensors of glutamate release that occurs during both moderate and strong neuronal activities [21, 53, 96].

Evidently, one of the many roles of astrocytes in the CNS is to take up excess neuroactive substances resulting from physiological neuronal transmission. This is fundamental to maintaining extracellular homeostasis and thus normal brain functions. Such processes are undoubtedly subject to stringent regulations and are elegantly compartmentalized in the brain. While they have been found to take part in the shaping of neuronal activities [45], the proposition that these processes are also plastic and may be neuronal activity-dependent is just becoming of interest. In the following sections, we focus on important research findings supporting this notion, while proposing possible molecular mechanisms that might be involved in making these types of astroglial plasticity possible.

## 5. Plasticity of Astroglial Potassium Clearance

In order to maintain proper brain homeostasis, the process of astroglial K^+^ clearance must also adapt to handle dramatic changes in extracellular K^+^ levels over different strengths of synaptic activity. Using electrophysiological techniques, there have only been a few studies demonstrating short- or long-term activity-dependent plastic changes in astroglial K^+^ uptake. Nevertheless, they uncover unique characteristics of astrocytic currents, which are of important value in the field. Thus, we summarize these research findings concentrating on the similarities and differences between astroglial and neuronal functional plasticity. We will also present novel findings to further illustrate the involvement of activity-dependent K^+^ clearance by hippocampal astrocytes during mild activities.

### 5.1. Short-Term Plasticity of Astrocytic Potassium Currents

**Figure 2:**
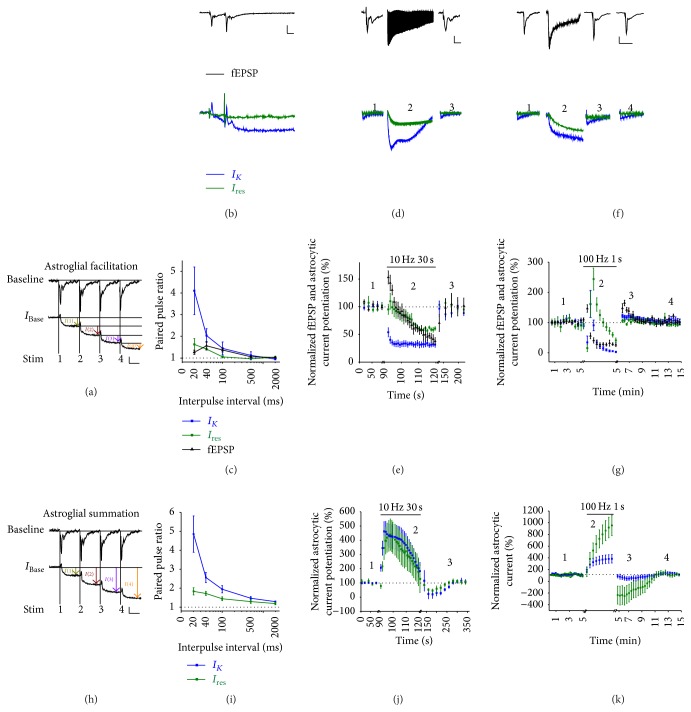
Short-term plasticity of astroglial potassium and residual currents quantified using two different methods. Simultaneous recordings of field excitatory postsynaptic potentials (fEPSPs) and synaptically evoked astroglial currents were carried out in hippocampal CA1 region upon stimulation of Schaffer collaterals. (a, h) Two different methods used to quantify astroglial currents are illustrated. fEPSPs (top) and astroglial currents (bottom) during the first four stimulations of a 10 Hz train are shown. The peak amplitude of each response was measured either in reference to (a) the prestimuli baseline, taken just before each stimulation (astroglial facilitation) or (h) to the initial resting baseline (*I*_Base_), taken before the first stimulation (astroglial summation). Scale bars = 0.05 mV for fEPSP, 20 pA for astroglial currents, 25 ms. (b, d, and f) Sample traces are shown for fEPSPs (top) and pharmacologically isolated astrocytic potassium *I*_*K*_ (bottom, blue) and residual *I*_res_ (bottom, green) currents in response to (b) paired-pulse stimulation (interpulse interval = 40 ms; scale bars = 0.5 mV for fEPSP, 20 pA for astroglial currents, 25 ms), (d) repetitive stimulation (10 Hz for 30 s; scale bars = 0.2 mV for fEPSP, 40 pA for astroglial currents; 5 s for astroglial currents and 50 ms for fEPSP), and (f) tetanic stimulation (100 Hz for 1 s; scale bars = 0.2 mV for fEPSP, 20 pA for astroglial currents; 7.5 s for astroglial currents and 60 ms for fEPSP). Quantifications using (c, e, and g) astroglial facilitation or (i, j, and k) astroglial summation methods are shown for (c, i) paired-pulse ratio, and normalized fEPSP slope and astrocytic current amplitudes during (e, j) repetitive or (g, k) tetanic stimulation. Numbers indicate extracted traces before (1), during (2), and after (3, 4) each stimulation. Mean ± SEM are shown for (c), (e), (g), (i), (j), and (k).

Hippocampal CA3-CA1 synapses exhibit several forms of short-term plasticity induced by distinct stimulation protocols of SCs, which are mediated by changes in presynaptic glutamate release probability (Pr) [21, 53, 96]. As we have previously reported, synaptically evoked astroglial currents in the hippocampus exhibit complex characteristics [21, 93, 97]. In particular, upon dissection of astrocytic current components, a long lasting Kir4.1 channel-mediated current (*I*_*K*_) and a residual current component (*I*_res_) could be identified. *I*_res_ is in part contributed by Kir4.1 channel-independent current. A classical type of short-term plasticity is paired-pulse facilitation (PPF), which is assessed by two successive stimulations with a short interstimulus interval (ISI) of 40 ms. An increase in the magnitude of the second postsynaptic response is thought to result from the residual increase of Ca^2+^ concentration in presynaptic terminals in response to the first stimulation. A common practice in electrophysiological measurements of neuronal-related response is to quantify the peak (or slope) of the response measured from the baseline immediately before the stimulation as illustrated in Figure 2(a) as the “astroglial facilitation” method. With this, it is evident that following paired-pulse stimulation at different frequencies (0.5 to 50 Hz), while neuronal field excitatory postsynaptic potentials (fEPSPs) potentiated more at an ISI of 40 ms, astrocytes exhibited a robust potentiation of *I*_*K*_, which was strongest at 20 ms ISI instead (Figures 2(b) and 2(c)). Similarly, *I*_res_ also potentiated but to a much lesser extent, similar to the level of fEPSPs. In addition to PPF, prolonged repetitive stimulation (10 Hz for 30 s) and tetanic stimulation (100 Hz for 1 s) of SCs are two other well-known stimulation protocols used to induce short-term plasticity. Repetitive stimulation induces fast and transient potentiation of neuronal fEPSPs, as a result of the massive release of glutamate, followed by rapid response depression, due to the depletion in glutamate vesicular pools (Figures 2(d) and 2(e)). The tetanic stimulation, on the other hand, is commonly used to induce posttetanic potentiation (PTP), a transient potentiation lasting a few minutes (Figures 2(f) and 2(g)). When astrocytic currents are quantified using the “astroglial facilitation” method, a rapid depression of both *I*_*K*_ and *I*_res_ was detected during the repetitive stimulation (Figure 2(e)). This was unexpected given the strong facilitation of these currents upon paired-pulse stimulation. Surprisingly, during the tetanus, both *I*_*K*_ and *I*_res_ initially potentiated much more strongly than during repetitive stimulation. This was followed by depression with *I*_res_ much more slowly than *I*_*K*_ (Figure 2(g)). Moreover, compared to the neuronal response, *I*_*K*_ and *I*_res_ only showed mild PTP immediately after the tetanus.

It is important to note that, due to the longer-lasting nature of astrocytic *I*_*K*_ and *I*_res_ current responses compared to neuronal fEPSPs, an incomplete recovery of the baseline before subsequent stimulations is often observed. Thus, such significant baseline shift must be taken into account during the quantification. To fully acknowledge this factor, it is therefore more appropriate to measure evoked current amplitude of each response from the initial baseline immediately before the first stimulation as illustrated in Figure 2(h). Indeed, this alternative method we define as “astrocytic summation” revealed very different and sometimes completely opposite short-term plastic behaviour upon repetitive and tetanic stimulations. During repetitive stimulation, both astrocytic *I*_*K*_ and *I*_res_ show short-term summation but with different kinetics to neuronal fEPSPs. Specifically, they initially summate up to ~400% (compared to ~150% potentiation in fEPSPs; Figure 2(e)), which lasted for 20 s and decreased back to baseline during the last 10 s of the stimulation (Figure 2(j)). This slower kinetics could be explained by K^+^ release from both pre- and postsynaptic elements leading to a uniform bulk increase of extracellular K^+^ level over larger zones requiring more time to be removed [23]. Such behavior has been previously reported in other brain regions and is thought to correspond to glial depolarization leading to extracellular K^+^ build-up over time, as originally described in the optic nerve [60] and subsequently in the cortex* in vivo* [98–100]. Interestingly, during a tetanic stimulation, greater distinctions between neuronal fEPSPs and astroglial currents were uncovered using this quantification method. While fEPSPs mildly potentiated during the initial phase of the tetanus (~150%; Figure 2(g)), astroglial *I*_*K*_ showed a stronger and longer-lasting summation (reaching ~400%), which continued during the course of the stimulation (Figure 2(k)). Remarkably, the *I*_res_ exhibited an even stronger summation (~1000%; Figure 2(k)) during the tetanus. Moreover, PTP observed in neuronal fEPSPs was not detected in either *I*_*K*_ or *I*_res_. In contrast, a posttetanic depression of current undershoot of mild (~−30%) and stronger (~−200%) magnitudes were detected in *I*_*K*_ and *I*_res_, respectively (Figure 2(k)). Similar undershoot response has been characterized following high-frequency stimulations [101] and could represent a period of redistribution of K^+^ in the extracellular space to avoid overdepletion. In addition, the astroglial facilitation quantification appeared to underestimate the undershoot currents rendering *I*_*K*_ and *I*_res_ mildly potentiating after tetanic stimulation. It is important to point out that PPF, quantified by comparing only two successive stimulations at a time, does not seem to differ significantly using the two methods (Figures 2(c) and 2(i)). With this, we stress the importance of using an appropriate quantification method, “astrocytic summation,” when measuring astrocytic slow currents, which takes into account baseline shifts over successive high-frequency stimulations.

**Figure 3:**
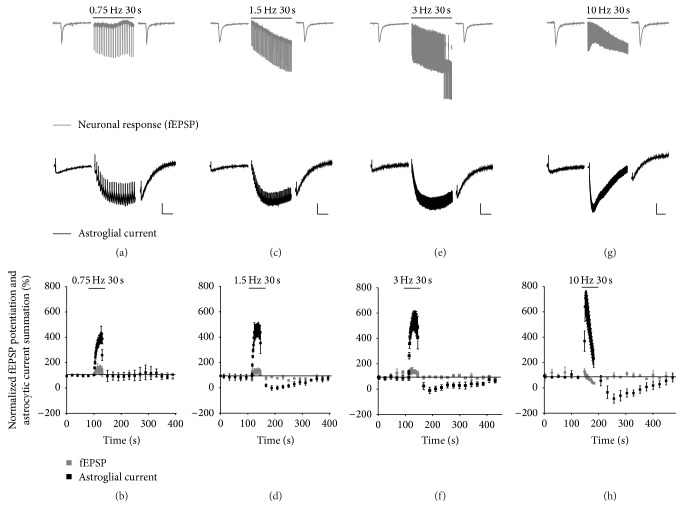
Short-term summation of astroglial currents compared to neuronal response during low frequency stimulations. Simultaneous recordings of field excitatory postsynaptic potentials (fEPSPs) and synaptically evoked astroglial currents were carried out in hippocampal CA1 region upon stimulation of Schaffer collaterals. (a, c, e, and g) Sample traces are shown for fEPSPs (top, gray) and astrocytic currents (bottom, black) in response to 30 s stimulations at indicated frequencies (0.75 to 10 Hz). Scale bars = 0.2 mV for fEPSP, 20 pA for astroglial currents; 4 s for baseline, 5 s during stimulations. (b, d, f, and h) Quantifications using astroglial summation method (see Figure 2) are shown for normalized fEPSP slope (gray) and astrocytic current amplitudes (black) in response to 30 s stimulations at indicated frequencies (0.75 to 10 Hz). Stimulations are denoted by bars above traces or quantifications. Mean ± SEM are shown for (b), (d), (f), and (h).

When comparing short-term plasticity of astrocytic currents and neuronal response, it is also important to consider their differential characteristics towards lower stimulation frequencies. For this reason, and given that ~80% of astroglial slow inward current (*I*_tot_) is composed of *I*_*K*_ [21], we measured astrocytic inward current *I*_tot_ in response to trains of SCs stimulation at a range of lower frequencies (0.75, 1.5, 3, and 10 Hz for 30 s) in hippocampal slices. Importantly, this was performed simultaneously with neuronal excitatory neurotransmission recordings in order to correctly assess both neuronal and astroglial (Figures 3(a), 3(c), 3(e), and 3(g)) short-term plastic behavior. As illustrated in Figure 3, neurotransmission exhibited weak short-term plasticity following 0.75–3 Hz stimuli for 30 s (with potentiation up to 120 and 150% at 1.5 and 3 Hz, resp.). Astroglial *I*_tot_, on the other hand, remarkably showed very strong summation of ~400% during 0.75 Hz stimulation (Figure 3(b)). This current was quantified using the “astrocytic summation” method as described above. Such summation was enhanced to ~500% during 1.5 Hz and even ~600% during 3 Hz stimulations (Figures 3(d) and 3(f)). In response to 10 Hz repetitive stimulations, a near 800% summation of astroglial *I*_tot_ was observed as previously reported (Figure 3(h)) [21]. In addition, such short-term summation exhibited longer-lasting kinetics than the corresponding neuronal response, which could be explained by the dominating *I*_*K*_ component in *I*_tot_, which is strongly influenced by the rate of K^+^ clearance from the extracellular space. Altogether, these data indicate that astroglial slow inward current short-term plasticity is also strongly involved at low frequencies of repetitive stimulations. More importantly, this frequency range is below what is usually used for neuronal short-term plasticity studies and induced only weak potentiation in neuronal fEPSPs. It can be postulated that short-term plasticity of astroglial K^+^ uptake observed at low frequencies may be a mechanism of activity-dependent upregulation of K^+^ buffering important for controlling neuronal responses.

### 5.2. Long-Term Plasticity of Astrocytic Potassium Currents

While LTP and LTD have been well established and described in neurons to be associated with learning and memory [102], the topic of astroglial long-term plasticity is yet to be characterised. Indeed, most of the existing studies concerning astroglial long-term plastic properties have concentrated on glutamate receptors [11, 15] and transporters [12, 16, 19, 20, 54, 96] (see Section 6), leaving K^+^ current-mediated plasticity virtually unexplored.

Nevertheless, there have been two important reports on changes in evoked astrocytic currents or potentials upon long-term synaptic potentiation. The first study by Ge and Duan [13] was carried out in the hippocampus CA1 region. Using electrophysiological techniques, the authors showed that tetanic stimulation (100 Hz for 1 s) of SCs evoked persistent LTP in fEPSPs of CA1 pyramidal neurons. This was accompanied by a LTP-like response in astroglial slow inward current resulting from membrane depolarization. Interestingly, similar to short-term plasticity of astroglial inward current described above, the astrocytic response exhibited stronger potentiation than the neuronal fEPSPs during both the initial and late phases of LTP. Furthermore, this astroglial LTP was sensitive to blockers of NMDA receptors and K^+^ channels, but not intracellular Ca^2+^ level in astrocytes. This suggested a mechanism involving postsynaptic NMDA receptor activation leading to accumulation of extracellular K^+^. Such increase in extracellular K^+^ level was estimated to be from 5.1 to 17.9% corresponding to an increase in astrocytic depolarization from 1 to 3 mV following LTP induction. The authors, however, postulated that the actual depolarization at peripheral astrocytic processes is likely to be even higher compared to the soma. Finally, it is important to note that although LTP of astroglial depolarization in this case may be a passive response, the resulting downstream astroglial responses may serve as persistent feedback signals in turn modulating neuronal behavior. Although not directly tested in this study, it is likely that such plasticity is largely mediated by astrocytic Kir4.1 channels as we have demonstrated that synaptically evoked astrocytic currents consisted of ~80% Kir4.1 channel dependent *I*_*K*_ current [21]. However, whether this long-term increase in K^+^ current is mediated by channel expression, localization or other factors remains unanswered. It is also possible that an accompanying decrease in glial glutamate uptake resulting from membrane depolarization plays a role in the progression of the astroglial LTP observed.

Building upon these findings, a later study reported by Zhang and colleagues [22] also described an LTP-like response in astrocytes, induced by strong high-frequency stimulation (HFS; 8 trains of 8 pulses at 200 Hz with intertrain interval of 2 s) of the perforant path in the dentate gyrus (DG) of the hippocampus. Indeed, a ~400% potentiation in astroglial membrane depolarization induced by HFS, referred to as excitatory postsynaptic astrocytic potentials (EPAPs) by the authors, was observed and lasted for more than 30 min. Importantly, the magnitude of this astroglial potentiation was more pronounced than that during neuronal LTP upon the same HFS stimuli. Next, it was demonstrated that neither glutamate transporters (GLT-1 and GLAST) nor metabotropic glutamate receptors (group I mGluRs) were responsible for the astroglial EPAPs potentiation, as LTP induction was unaffected by treatments with DHK (GLT-1 inhibitor), threo-*β*-hydroxyaspartic acid (THA; a GLAST inhibitor), or dihydroxyphenylglycine (DHPG; a group I mGluR agonist). However, by combining astrocytic EPAPs recordings with single-cell reverse transcription-polymerase chain reaction (RT-PCR), the authors elegantly showed that LTP induction required functional NMDA receptor activation in astrocytes at the granule cell body layer of the DG. Indeed, significant PCR products for NMDAR subunits (NR1, NR2A, and NR2B) were also detected, in agreement with previously characterised functional astroglial NMDA receptors on astrocytes [103]. Altogether, even though this study did not directly investigate astroglial K^+^ currents, it is likely that, similar to the first study [13], the NMDA receptor-mediated astroglial LTP observed here is also associated with changes in extracellular K^+^ increase.

In summary, although astrocytes are sensitive to and respond quickly to changes in extracellular K^+^ levels, not much is known about how this response is regulated in a neuronal activity-dependent manner in terms of both short-term and especially long-term plasticity. Nevertheless, these few available studies provided encouraging first evidence of the existence of such form of plasticity, as well as how they can be appropriately characterised. With this, it is important to focus future research on detailing the underlying mechanisms in terms of identifying channel subtypes and regulating factors involved, as well as the interconnection with other types of astroglial plasticity like glutamate uptake.

## 6. Plasticity of Astroglial Glutamate Clearance

While one of the main functions of astrocytes is to remove excess glutamate during excitatory neurotransmission, it is of interest to know if astroglial glutamate clearance also exhibits different forms of short- and long-term plasticity in order to adapt to changing neuronal activities. In particular, several studies have focused on electrophysiological measurements of astroglial synaptically-activated glutamate transporter currents (STCs) as a means of determining glial glutamate transporter activities. Remarkably, both short- and long-term plasticity have been documented either in synchrony with or opposite to changes in synaptic transmission. Here, we review these studies focusing on the plasticity of astroglial STCs and discuss their roles in various experimental models and brain areas.

### 6.1. Short-Term Plasticity of Astroglial STCs

**Figure 4:**
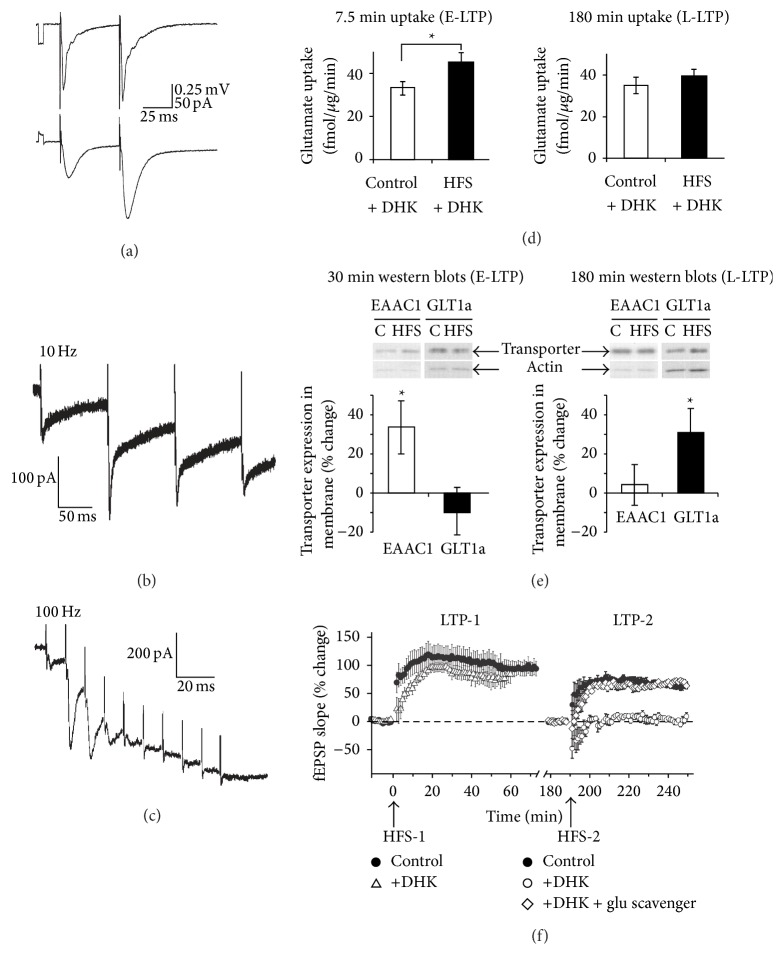
Short- and long-term plasticity of astroglial glutamate uptake. (a) Simultaneous recordings of neuronal responses (fEPSPs; field excitatory postsynaptic potentials) and astroglial glutamate transporter currents (STCs) revealed paired-pulse facilitation of both fEPSPs and astroglial glutamate uptake in hippocampal CA1 region. Sample traces are shown for fEPSPs (top) and astroglial STCs (bottom). STCs were recorded in the presence of 5 mM kynurenic acid. Scale bars = 0.25 mV for fEPSP, 50 pA for astroglial currents, 25 ms. (b-c) Patch-clamp recordings revealed short-term facilitation of astroglial extrasynaptic currents (ESCs) in the cerebellum. Sample traces are shown for ESCs in response to 10 pulses of (b) 10 Hz or (c) 100 Hz stimulation to parallel fibers. (d-e) Astroglial GLT-1 glutamate transporter is responsible for glutamate uptake during late but not early phase of long-term potentiation (LTP) in the hippocampus. (d) Bar graphs showing that the GLT-1 inhibitor, dihydrokainate (DHK), inhibited glutamate uptake during late phase (L-LTP; right panel), but not early phase (E-LTP; left panel) of LTP induced by high frequency stimulation of SCs (HFS; each composed of two trains of 100 Hz pulses separated by 20 s). Glutamate uptake in hippocampal slices were measured using radioactive glutamate (L-[14C(U)]-glutamic acid). (e) Western blot analysis revealed a significant upregulation of neuronal glutamate transporter (EAAC1) expression during E-LTP (left panel) and of GLT-1 during L-LTP (right panel). Representative western blots are shown above bar graphs. (f) Recordings of fEPSPs in hippocampal CA1 region showed that an increase in GLT-1 activity and glutamate uptake are required for the induction of additional LTP. LTP was triggered twice in the same experiment (LTP-1 and LTP-2). Each LTP was induced by HFS as indicated by arrows (HFS-1 and HFS-2). During LTP-1, no difference was observed between slices treated with DHK (open triangles) and control (filled circles) indicating that basal GLT-1 activity was not required for induction of LTP-1. After the establishment of a new baseline, an additional LTP (LTP-2) was triggered under control conditions (filled circles). This was completely inhibited in slices treated with DHK (open circles). The effect of DHK was abolished in the presence of a glutamate scavenger (glutamic-pyruvic transaminase + pyruvate). These suggested that an increase in GLT-1 activity above basal level and the accompanying enhanced glutamate uptake is important for the induction of LTP-2. Mean ± SEM are shown for (d–f). Adapted with permission: (a) [12], (b-c) [10], (d-e) [19], and (f) [20].

Similar to the studies of astroglial *I*_*K*_ discussed above, electrophysiological measurements using the same stimulation protocols can also be applied to characterize short-term plasticity of astroglial STCs. With PPF protocols, it was observed by Diamond and colleagues [12] that astroglial STCs remarkably displayed a 1.9-fold PPF (ISI = 50 ms) similar to the 1.4-fold PPF of AMPA receptor-mediated EPSCs in CA1 pyramidal neurons (Figure 4(a)). This supported the notion that glial transporter currents accurately monitored glutamate release at CA3-CA1 synapses [12, 21, 53, 93, 94, 96].

Paired-pulse ratio (PPR) of glial STCs, in contrast to AMPA-EPSCs, was however not directly connected to the Pr, as manipulation of Pr by either changing extracellular Ca^2+^ concentration or blocking adenosine A1 receptors did not induce any change in glial PPF [104]. In the cerebellum, Bergmann cells are specialized glial cells, which interestingly express both glutamate transporters and GluA2-lacking AMPA receptors that are permeable to Ca^2+^ [105, 106]. With this in mind, the authors measured a striking 4.2-fold PPF of extrasynaptic currents (ESCs) induced by parallel fibers paired stimulations [10], whereas a similar protocol applied to Purkinje neurons only induced a 2-fold increase [107]. Upon isolation of glutamate uptake components from the ESCs, a 1.8-fold PPF of glial STCs similar to synaptic PPF resulted [10]. PPF has also been detected in both medium-sized spiny neurons, sensors of cortical activity, and glial STCs following stimulation of layer 5 of the somatosensory cerebral cortex [14]. Interestingly, for ISIs of 4 to 10 ms, while cortical STCs still displayed PPF, EPSCs exhibited depression. The authors postulated that these opposite observations could be due to a facilitation of glial glutamate uptake leading to decreased glutamatergic postsynaptic activation.

Apart from PPF, glial response to repetitive stimulation (10 Hz for 30 s) was also investigated. With such stimulation protocol, it was demonstrated that astroglial STCs, in synchrony with neuronal responses, first potentiated and then depressed with a similar time course to that of fEPSPs in the hippocampus [21]. In the cerebellum, repetitive stimulation of parallel fibers even at low frequencies (0.1 to 1 Hz) displayed initial glial ESCs facilitation similar to SCs stimulation followed by depression [10], suggesting that distant Bergmann glial processes are in a more depolarized state than the soma (Figure 4(b)).

Finally, similar to fEPSPs generated by neurons, astroglial STCs also initially potentiated and then depressed in response to 100 Hz tetanus stimulation for 1 s [10, 21]. Furthermore, a train of 10 pulses at 100 Hz stimulation of cerebellum parallel fibers generated facilitation of glial ESCs amplitude that gradually decreases and subsequently turned to depression (Figure 4(c)). Taken together, these studies indicate that hippocampal, cortical, and cerebellar astroglial STCs displayed a neuronal-like plasticity and monitored short-term excitatory synaptic transmission by reflecting activities of surrounding synapses.

### 6.2. Long-Term Plasticity of Astroglial STCs

As astroglial STCs exhibit various short-term plastic changes induced by modifications of neurotransmitter Pr, it is also of interest to investigate whether such plasticity can also be long-lasting. Indeed, using paired granule cell-glial cell recordings in cerebellar cultures, LTP of Ca^2+^-permeable AMPA receptor glial currents has been induced by prolonged 4 Hz stimulation of the granule cell [15, 16]. This provided one of the first evidence showing that astroglial glutamate transporter currents, in addition to neuronal transporters, are able to display LTP. LTP of glial transporter currents could be induced in the presence of postsynaptic Ca^2+^ chelation and glutamate receptor antagonists but was blocked by removal of external Ca^2+^ during the tetanus, suggesting that a part of the cerebellar LTP is expressed presynaptically through modifications of glutamate Pr [16].

Over the years, many insights regarding astroglial glutamate uptake have been obtained from neuronal STCs displaying long-term plasticity. An important study on neuronal glutamate transporters (EAAT4) in cerebellar Purkinje cells has shown that STCs undergo LTP in response to brief tetanic stimulation of climbing fibers [108]. Importantly, the increase in neuronal glutamate transporter current was coupled to LTD of AMPA-EPSCs, suggesting the involvement of an integrated mechanism that protects Purkinje cells from glutamate excitotoxicity. Other studies have shown that neuronal and astroglial glutamate transporters have distinct roles during long-term changes in neuronal activities. In the hippocampal CA1 area, it was observed that EAAC1, the predominant neuronal glutamate transporter, is upregulated during the early phase of LTP (E-LTP) and fear conditioning* in vivo* [109]. The enhanced glutamate uptake accompanying the increased surface expression of glutamate transporters was the likely mechanism limiting glutamate spillover during E-LTP. As neuronal EAAC1 and glial GLT-1 together account for more than 80% of glutamate uptake in the hippocampus [110], the changes in GLT-1 expression and activity was also investigated in this study. To assess differential contributions of these two transporters, the authors showed that the increase in glutamate uptake during E-LTP, observed together with an increase in EAAC1 expression, did not require macromolecular synthesis, and was not sensitive to DHK, a selective inhibitor of GLT-1. This increase in glutamate uptake that occurred at the postsynaptic element could prevent postsynaptic glutamate receptor desensitization during strong neuronal activity or limit glutamate spillover. Although glial GLT-1 activity was not altered during E-LTP, a later study revealed an increase in glial glutamate uptake during the late phase of LTP (L-LTP), which could be explained by an upregulation of GLT-1 expression at the membrane resulting in increased astroglial glutamate uptake (Figures 4(d) and 4(e)) [19]. This was found to be sensitive to protein synthesis and transcription inhibition, as well as DHK, and may participate in controlling both presynaptic release and extracellular diffusion of glutamate. Additionally, the same group later demonstrated that an increase in GLT-1 activity above basal level, and the accompanying enhancement in glutamate uptake, appears to be responsible for the induction of additional LTP (Figure 4(f)) [20]. This interesting discovery demonstrated the importance of long-term upregulation of astroglial glutamate uptake in the maintenance of synaptic plasticity. These studies together supported differential regulations of both neuronal and glial glutamate transporters during the progression of LTP. Finally, Bergmann glia AMPA receptors and glutamate transporters have been found to display activity-dependent LTD during parallel fibers stimulation. Bellamy and Ogden [11] revealed that this plasticity of cerebellar glial cells was, however, not observed in synchrony with surrounding neuronal activity. Indeed, LTD of glial ESCs and Ca^2+^-permeable AMPA receptor currents was induced in Bergmann glia using a 0.2 Hz frequency stimulation that does not generate any change in evoked excitatory postsynaptic current (EPSC) amplitude from Purkinje neurons.

Taken together, these results revealed that astrocytes are sensitive to different forms of plasticity. Long-term plasticity of both neuronal and astroglial STCs are essential regulators of neuronal homeostasis. Therefore, by controlling presynaptic neurotransmitter release, as well as extracellular diffusion of glutamate to prevent postsynaptic receptors desensitization, these long-term mechanisms are necessary to protect the tripartite synapse against glutamate excitotoxicity that occurs during sustained neuronal activity (e.g., neurological disorders such as epilepsy [111], addiction [112], and neurodegenerative diseases [113, 114]).

### 6.3. Possible Mechanisms Mediating Long-Term Plasticity of Astroglial STCs

While it is evident that astrocytes display both short- and long-term plasticity in glutamate uptake, a description of the possible molecular mechanisms underlying such activity-dependent regulation remains unexplored and still needs to be clarified. Interestingly, and due to its simple nervous system organization, the marine mollusk* Aplysia californica* has been used as a useful model to study changes in neural circuitry that occurred during specific behaviour like long-term sensitization (LTS) [115, 116]. Similar to later discoveries in the CA1 region [109], enhanced glutamate uptake was triggered by* in vivo* treatment with serotonin or electrical stimulation, which are responsible for the induction of LTS in* Aplysia*. Importantly, the PKA, MAP, and tyrosine kinase pathways appeared to regulate glutamate uptake by either transcription of the glutamate transporter or posttranslational modifications such as phosphorylation [82, 117, 118]. In addition, the primary high affinity glutamate transporter in* Aplysia*, ApGT1, is transported through the endoplasmic reticulum-Trans-Golgi network vesicular trafficking system from the sensory neuron somata to the terminals. This pathway may be the mechanism underlying the increase in ApGT1 at the membrane, and consequently leading to glutamate uptake observed during long-term plasticity [119, 120]. Taking advantage of the similarities in learning and memory behaviour between* Aplysia* and higher vertebrates [121], these proposed pathways give important insights into the molecular mechanisms governing plasticity of glutamate uptake in glial cells. In fact, the importance of glial glutamate transporter trafficking has already been under consideration. By using a combination of high-resolution live imaging and electrophysiological recordings both* in vitro* and* in vivo*, a recent study [92] provided additional evidence regarding the role of astrocytic GLT-1 surface trafficking in shaping excitatory synaptic transmission. Noteworthy, the diffusion coefficient of GLT-1 was one of the highest reported while the fraction of immobile transporters was one of the lowest. These findings, together with an earlier study [122], clearly suggest that scaffolding and/or anchoring proteins may directly control transporter surface diffusion. Additional studies focused on the identification of both neuronal and glial glutamate transporter binding partners will further clarify how short- and long-term changes in glutamate transporter could occur during neuronal activity.

### 6.4. Morphological Plasticity in Astrocytes

Apart from short- and long-term plasticity of glutamate uptake, astrocytes also display changes in their morphology in response to sensory stimuli or neuronal activity [14, 17, 123, 124]. Indeed, it has recently been reported that LTP transiently increases the motility of hippocampal perisynaptic astrocyte processes, which is mediated by calcium signalling induced by activation of metabotropic receptors. In addition, structural plasticity of astroglial processes has also been observed in the somatosensory cortex following whisker stimulation [123, 124].

Remarkably, such morphological plasticity has been described to modify glutamate transporter activities in several brain areas. One of the first evidences was identified during lactation, a physiological intense stimulation. Oliet and colleagues [17] have observed that glutamate level in the synaptic cleft was higher in lactating animals than virgin or postlactating animals and correlated it to a reduced astrocytic coverage onto supraoptic nucleus (SON) neurons. In addition, inhibition of SON glutamate uptake by DHK consequently resulted in glutamate spillover, which activated presynaptic group III mGluRs leading to a lower glutamate Pr. Few years later, using single whisker stimulation paradigm, which increases sensory activity, another group showed an increase in GLT-1 and GLAST protein expression in barrels corresponding to the stimulated whisker [14]. Notably, morphometric analyses using serial electron microscopy confirmed an increase in astroglial coverage to the bouton-spine interface, suggesting that plasticity of glutamate transporters in the somatosensory cortex may prevent glutamate spillover to surrounding areas. Finally, we have shown that hippocampal astrocytes display Cx30-mediated morphological changes, which in turn regulates astroglial glutamate uptake [18]. It was found to be mediated by nonchannel functions of Cx30. In particular, an increase in the insertion of astroglial processes into synaptic clefts was observed in Cx30 deficient mice. Such increased invasion of excitatory synapses could facilitate glutamate uptake observed in these mice. Together, these important studies concurrently demonstrated enhanced synaptic coverage by astrocytic processes with increased glutamate uptake via astroglial glutamate transporters in different brain regions. This implies that astrocytes may take advantage of the positioning of their dynamic processes to regulate glutamate removal from the synaptic cleft.

## 7. Conclusions and Perspectives

Astrocytes not only sense but also respond to neuronal activities and have been recognized as active players at the tripartite synapse. Over the years, it has been of immense interest for researchers to explore such intricate relationships between neurons and astrocytes. In particular, among many astroglial functions, their abilities to remove excess K^+^ and glutamate upon neuronal activity are thought to be essential mechanisms important for the maintenance of extracellular homeostasis and thus allow proper functioning of the brain. For this very purpose, astrocytes express functional K^+^ channels as well as glutamate transporters. With the help of their elaborate gap-junctional networks, they have been found to efficiently take up and redistribute excess neuroactive substances throughout the brain. Interestingly, over recent years, the focus has extended to activity-dependent plasticity of these properties. It was hypothesized that in response to changes in synaptic strengths, these astroglial properties can be modulated in an activity-dependent manner, similar to neuronal synaptic plasticity. Indeed, both short- and long-term plasticity of such nature have been documented as presented in this review. It is important to note that in some cases, astroglial responses behave in concert with neurons [10, 12, 21]. However, in other cases, they exhibit unique kinetics [21] and differential regulations [19, 20] as compared to their neuronal counterparts. Importantly, we have also shown here that astrocytic inward currents display robust summation during mild-stimulations, suggesting differential sensitivity to the surroundings by neurons and astrocytes.

With these emerging findings, we show in this review that astroglial properties indeed display different forms of plasticity in response to changing neuronal activities. However, the exact cellular and molecular mechanisms mediating such processes still remain elusive. Nevertheless, it has been postulated that channel and transporter expression, clustering, and trafficking might play important roles [19, 92, 122]. Moreover, astrocytes may also undergo morphological changes in synaptic coverage by their fine processes, thereby modulating uptake [14, 17, 18]. In order to better understand activity-dependent astroglial plasticity and how it may be triggered and regulated, it is essential to explore the underlying cellular and molecular mechanisms governing these changes. It is with this knowledge of plasticity from both neuronal and glial points of view, and how they are interconnected, that we can fully appreciate the dynamic aspects of brain processes like learning and memory.
